# Enhanced Secretary Bird Optimization Algorithm for Energy-Efficient Cluster Head Selection in Wireless Sensor Networks

**DOI:** 10.3390/s26051732

**Published:** 2026-03-09

**Authors:** Ketty Siti Salamah, Dadang Gunawan, Ajib Setyo Arifin

**Affiliations:** Department of Electrical Engineering, Universitas Indonesia, Depok 16424, Indonesia; ketty.siti@ui.ac.id (K.S.S.); ajib.sa@ui.ac.id (A.S.A.)

**Keywords:** wireless sensor networks, energy-efficient clustering, cluster head selection, metaheuristic optimization, secretary bird optimization algorithm

## Abstract

Cluster Head (CH) selection is a crucial process in clustered Wireless Sensor Networks (WSNs) because it directly affects energy balance and network lifetime. However, CH selection is an NP-hard optimization problem, and many metaheuristic-based methods suffer from limited search diversity and premature convergence, leading to uneven energy dissipation. This paper formulates CH selection as a multi-criteria energy-aware optimization problem and proposes an Enhanced Secretary Bird Optimization Algorithm (ESBOA). The proposed ESBOA improves the original Secretary Bird Optimization Algorithm by integrating logistic chaotic map-based population initialization to enhance early-stage exploration and an iterative local search mechanism to strengthen solution refinement in later iterations. A multi-criteria fitness function considering residual energy, distance to the base station, and node degree explicitly guides the optimization toward energy-efficient clustering. The proposed method is implemented in a Python 3.11.9-based simulation framework using a first-order radio energy model and evaluated against standard SBOA, Crested Porcupine Optimization (CPO), and Dung Beetle Optimization (DBO). Simulation results demonstrate that ESBOA preserves more alive nodes, maintains higher residual energy, delivers more cumulative packets to the base station, and extends network lifetime, achieving approximately 3–13% improvement in last node death (LND) compared with the standard SBOA.

## 1. Introduction

**Figure 1:**
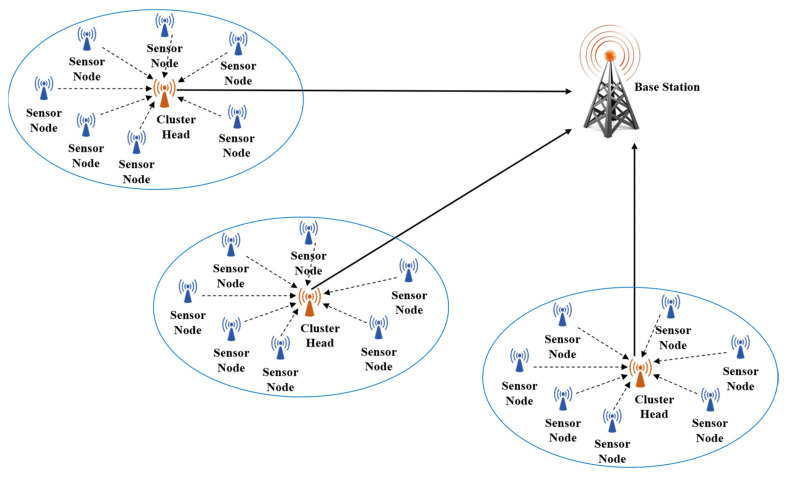
Conceptual architecture of a clustered WSNs.

Cluster Head (CH) selection plays a decisive role in clustered Wireless Sensor Networks (WSNs), as it directly affects energy consumption balance, network lifetime, and communication reliability [1]. As illustrated in Figure 1, the clustering process divides the network into smaller subgroups led by a CH, which significantly reduces communication overhead among nodes, especially in large-scale networks [2]. In cluster-based approaches, the sensors do not need to communicate directly with the base station. Instead, the CH is responsible for organizing cluster members and sending the data collected within the cluster to the base station [3,4]. In clustered WSN architectures, this hierarchical communication structure reduces the number of long-distance transmissions and improves scalability and network manageability. Clustering helps mitigate excessive energy consumption caused by direct node-to-base station communication by localizing data collection and forwarding tasks within clusters. Clustering is widely regarded as an effective strategy for prolonging network lifetime and supporting energy-aware communication, particularly in dense and large-scale sensor deployments [5].

However, the effectiveness of clustering strongly depends on the selection of an appropriate cluster head, as these additional aggregation and long-range transmission duties impose a disproportionate energy burden on CH nodes. Consequently, CH selection is widely recognized as a challenging non-deterministic polynomial (NP)-hard optimization problem [6], as it involves selecting an optimal subset of nodes while simultaneously considering multiple conflicting criteria such as residual energy, communication distance, and node distribution. To address the complexity of these NP-hard problems, various computational optimization techniques have been widely employed [7]. Numerous optimization strategies have been proposed to enhance network performance by optimizing key components, including BS placement, routing, and CH selection [8,9]. Metaheuristic methods have emerged as a promising solution for addressing optimization challenges in WSNs. These methods fall into the category of optimization techniques designed to handle problems with high computational complexity. Metaheuristic approaches aim to efficiently find near-optimal solutions, especially in complex optimization problems that are difficult to solve exactly. The main advantages of these methods include ease of implementation, speed in generating solutions, and reliability in handling diverse problem characteristics [10].

Although various metaheuristic-based methods have been introduced to address CH selection, they often exhibit several practical limitations. Many approaches rely on random population initialization, which can reduce population diversity and increase sensitivity to initial conditions. Furthermore, an inadequate balance between exploration and exploitation may trap the search process in local optima, resulting in suboptimal CH configurations and uneven energy consumption. In addition, the lack of effective solution refinement mechanisms can lead to slow or unstable convergence, requiring excessive iterations and increasing computational overhead. In light of these challenges, including the NP-hard nature of CH selection and the limitations of existing metaheuristic approaches, swarm intelligence-based optimization methods have been widely adopted to improve the CH selection process [11,12]. To enhance energy efficiency and extend the operational lifespan of WSNs, this study proposes an Enhanced Secretary Bird Optimization Algorithm (ESBOA) for CH selection in clustered WSNs.

The main contributions of this paper are as follows:

- The early-stage diversity of the NP-hard CH selection search space is often limited by random population initialization in metaheuristic-based CH selection methods. To address this issue, the ESBOA population is initialized using a logistic chaotic map, enabling more consistent exploration and improved search stability.
- Many swarm optimizers exhibit weak exploitation capability in later iterations, which can lead to premature convergence and suboptimal CH configurations. To overcome this limitation, an Iterative Local Search (ILS) mechanism is incorporated into ESBOA to enhance local exploitation and convergence stability.
- A multi-criteria fitness function considering residual energy, distance to the base station, and node degree is formulated to support energy-efficient clustering and balanced energy dissipation.

The remainder of this paper is organized as follows: Section 2 reviews the related work relevant to this study. Section 3 presents the proposed Enhanced Secretary Bird Optimization-based clustering framework, including the baseline Secretary Bird Optimization Algorithm, the proposed enhancements with adaptive population initialization and a local search refinement mechanism, the multi-criteria fitness function for cluster head selection, and the pseudocode of the ESBOA-based method. Section 4 reports the simulation results and discussion, covering the simulation settings, the homogeneous network model, and performance evaluation in terms of alive nodes, residual energy, network lifetime, and cumulative packets delivered to the base station. Finally, Section 5 concludes the paper and outlines potential directions for future work.

## 2. Related Works

Clustering is widely recognized in WSNs as a low-cost strategy that supports network scalability, maintains low latency, extends network lifetime, and minimizes energy consumption [13,14]. In the clustering mechanism, nodes are grouped into several clusters; one node in each cluster is selected as the CH, while the other nodes act as cluster members [15]. The CH collects data from cluster members, removes redundancy through aggregation, and then forwards relevant information to the BS, which is generally located further away. Consequently, CH energy consumption is greater because it must handle reception, aggregation, and long-distance transmission. Thus, CH selection is a key factor in balancing energy load distribution and extending network lifetime.

The CH selection problem remains classified as NP-hard, so achieving optimal network performance is still a complex problem. Therefore, metaheuristics are needed to optimize the critical parameters that influence CH selection decisions [16]. Swarm intelligence–based metaheuristic algorithms, such as Artificial Bee Colony (ABC) [17] and Particle Swarm Optimization (PSO) [12], have been widely applied to optimization problems in Wireless Sensor Networks. Chaurasia et al. [18] proposed a cluster-based routing scheme for WSNs by utilizing a metaheuristic approach focused on energy efficiency. In the study, the Dragonfly Algorithm (DA) is applied to determine the CH as well as the optimal communication path. The exploitation mechanism in DA is used in the CH selection process by considering parameters such as residual energy, distance, and node density, while the exploration strategy is implemented through a Lévy distribution-based random walk function for path determination. In addition, this DA-based clustering approach also takes into account the centrality aspect of nodes to support more reliable network communication. Dattatraya et al. [19] introduced the Fitness-based Glowworm- Fruit Fly (FGF) algorithm to enhance the effectiveness of the CH selection process. The FGF algorithm combines the principles of Glowworm Swarm Optimization with Fruit Fly Optimization to determine the optimal CH. In this scheme, the selection of CH is carried out by considering several key parameters, namely energy, latency, and distance between nodes. Subramanian et al. [20], the authors introduce CHS-EAWSN-TSO as a CH selection strategy designed to improve energy efficiency in WSNs. This approach utilizes the Transient Search Optimization (TSO) algorithm and emphasizes the determination of the optimal CH through a multi-criteria-based fitness function. The function integrates several important parameters, including the distance between nodes, residual energy, and transmission latency. The simulation results show that CHS-EAWSN-TSO provides better performance compared to conventional approaches. Recently, several swarm-inspired metaheuristics, including Crested Porcupine Optimizer (CPO) [21] and Dung Beetle Optimizer (DBO) [22], have been introduced to address complex and nonlinear optimization problems. Subramanian et al. [23] proposed a Hybrid Grasshopper Optimization Algorithm (GOA) and Crow Search Algorithm (CSA) based optimal CH Selection (HGWCSOA-OCHS) scheme, which is a hybrid approach that combines the Grasshopper Optimization Algorithm (GOA) and Crow Search Algorithm (CSA) to obtain optimal CH selection. This method is designed to extend network lifetime by reducing delay, inter-node distance, and maintaining energy consumption stability. In addition, this hybrid approach is intended to overcome premature convergence that can hinder effective search space exploration. Simulation results show a decrease in energy consumption and an increase in network lifetime, which is evaluated through the percentage of sensor nodes that are still active and those that have died within the network.

Bezhadi et al. [24] proposed a multi-objective energy-efficient clustering protocol that combines the Improved Binary Dragonfly Algorithm (IVBDA) for CH selection (based on residual energy, distance to BS, and neighborhood degree) and the Mamdani Fuzzy Inference System (FIS) for cluster formation (based on linguistic variables such as CH energy, node–CH distance, and CH neighborhood degree), and demonstrated improved performance and faster convergence compared to metaheuristic comparators such as BPSO, BWOA, and BDA, especially when IVBDA is enhanced with chaos mapping and improved local search. Current research also emphasizes the importance of strengthening internal metaheuristic components, particularly in the population initialization and solution refinement stages. Alshammri [25] developed the Improved Squirrel Search Algorithm (I-SSA) for CH selection by adding adaptive population initialization, dynamic step size control, and local search, as well as an objective function that considers CH balance, distance to BS, residual energy, and intra-cluster distance, thereby reducing energy consumption and cluster formation time and increasing metrics such as PDR and lifetime compared to several comparative methods. These findings confirm that practical issues that often arise in metaheuristics, namely random initialization, which can reduce the diversity of initial solutions, and the tendency to get stuck in local optima due to weak local exploitation, are still limiting factors in solution quality and convergence stability. Accordingly, strengthening population initialization and integrating local search are logical foundations for producing more stable and energy-efficient CH selection. Based on this gap, this study chose the Secretary Bird Optimization Algorithm [26] as the basis for optimization because its exploration-exploitation characteristics are suitable for searching for CH combinations that are close to optimal in a large solution space. However, like other metaheuristics, SBOA also has the potential to experience a decrease in initial solution diversity and stagnation at local optima when population initialization is still random and the solution refinement mechanism is limited. Therefore, this study proposes ESBOA with adaptive population initialization and local search reinforcement as well as multi-criteria fitness-based evaluation to improve convergence stability and energy efficiency in the CH selection process.

## 3. Enhanced Secretary Bird Optimization-Based Clustering

The CH selection process is a critical factor in achieving energy-efficient clustering in WSNs, as it directly affects energy consumption balance, network lifetime, and data transmission efficiency. Due to the combinatorial and NP-hard nature of the CH selection problem, metaheuristic optimization algorithms are well suited to search for near-optimal clustering solutions. The secretary bird optimization algorithm (SBOA) provides a flexible balance between exploration and exploitation; however, its direct application to CH selection may lead to limited search diversity and premature convergence. To overcome these limitations, this study proposes an enhanced secretary bird optimization algorithm (ESBOA)–based clustering approach that integrates adaptive population initialization and local search refinement mechanisms. In addition, a multi-criteria fitness function is formulated to guide the optimization process explicitly toward energy-efficient CH selection. The following subsections describe the SBOA framework, the proposed enhancements, the multi-criteria fitness function, and the complete ESBOA-based CH selection procedure.

### 3.1. Secretary Bird Optimization Algorithm

The secretary bird optimization algorithm (SBOA) is a nature-inspired metaheuristic motivated by the hunting strategies and survival mechanisms of the secretary bird, aiming to balance global exploration and local exploitation in complex optimization tasks [26]. The algorithm consists of two main phases: an exploration phase that models the hunting strategy of the secretary bird (including searching, consuming, and attacking prey) and an exploitation phase that models the escape strategy against predators. The initial implementation of SBOA utilizes Equation (1) as a random initialization scheme to determine the initial position of the secretary bird in the search space.

(1) $$X_{i,j}={lb}_{j}+r\times ({ub}_{j}-{lb}_{j})\;,i=1,2,\ldots ,N,j=1,2,\ldots ,Dim$$

where *X_i_* represents the position vector of the i-th secretary bird, while $lb_{j}$ and $ub_{j}$ denote the lower and upper bounds of the j-th dimension, respectively. r is a uniformly distributed random number in the range [0, 1].

The position update mechanism in SBOA is derived from two representative behaviors of the secretary bird, namely its hunting behavior and escape behavior.

(i) Hunting behavior in the exploration stage

In SBOA, the prey-hunting procedure is structured into three stages: searching prey, consuming prey, and attacking prey. During the prey-searching stage, each secretary bird updates its position according Equation (2):

(2) $$\mathrm{While}\;t<\frac{1}{3}\;T,\;\;X_{i,j}^{\mathrm{new}\;E1}=\;X_{i,j}+(X_{\mathrm{random}\_1}\;-\;X_{\mathrm{random}\_2})\times \;R_{1}$$

(3) $$X_{i}=\{\begin{array}{c} X_{i}^{\mathrm{new},E1},\;if\;F_{i}^{\mathrm{new},E1}<F_{i}\\ X_{i},\;\mathrm{else} \end{array}$$

where *t* denotes the current iteration index and *T* represents the maximum number of iterations. $X_{i}^{\mathrm{new},E1}\;$ refers to the updated position of the i-th secretary bird during the first stage, while *X_random_1_* and *X_random_2_* denote randomly selected candidate solutions at this stage. *R_1_* is a randomly generated vector of size 1 × *Dim* with values in the interval [0, 1], where *Dim* indicates the dimensionality of the solution space. $X_{i,j}^{\mathrm{new}\;E1}$ represents the *j*-th component of the updated solution, and $F_{i}^{\mathrm{new},E1}$ corresponds to the fitness value obtained from the objective function.

In the hunting strategy (exploration phase), three stages are considered: searching prey, consuming prey, and attacking prey. In the consuming prey phase, the update rule is defined as:

(4) $$\mathrm{While}\;\frac{1}{3}T\;<t<\frac{2}{3}T,\;X_{i,j}^{\mathrm{new}\;E1}=\;X_{\mathrm{best}}+\exp\;\left({\left(\frac{t}{T}\right)}^{4}\right)\times (X_{\mathrm{best}}\;-\;X_{i,j})\times (RB\;-\;0.5)$$

(5) $$X_{i}=\{\begin{array}{c} X_{i}^{\mathrm{new},E1},\;if\;F_{i}^{\mathrm{new},E1}<F_{i}\\ X_{i},\;\mathrm{else} \end{array}$$

In this stage, the position update is driven by Random Brownian (*RB*) motion to introduce stochastic perturbations and enhance local exploration. The individual historical best position $X_{\mathrm{best}}$ is incorporated to guide the search toward previously identified high-quality solutions. This mechanism balances exploration and exploitation, improves convergence efficiency, and mitigates premature convergence to local optima. The updated position is accepted only if it results in an improved fitness value.

In the attacking prey phase, a Lévy flight–based update strategy is employed to enhance global exploration and mitigate stagnation in local optima, as adopted in the original SBOA framework [26]. A nonlinear perturbation factor ${\left(1-\frac{t}{T}\right)}^{\left(\frac{2t}{T}\right)}$ is incorporated to adaptively balance exploration and exploitation during the later iterations. The combination of long jumps and short steps improves convergence accuracy and search efficiency. The updated position is accepted only if it yields an improved fitness value.

(6) $$\mathrm{While}\;t>\frac{2}{3}\;T,\;X_{i,j}^{\mathrm{new}\;E1}=X_{\mathrm{best}}+0.5\left({\left(1-\frac{t}{T}\right)}^{\frac{2t}{T}}\right)\times X_{i,j}\times \mathrm{Levy}(\mathrm{Dim})$$

(7) $$X_{i}=\{\begin{array}{c} X_{i}^{\mathrm{new},E1},\;if\;F_{i}^{\mathrm{new},E1}<F_{i}\\ X_{i},\;\mathrm{else} \end{array}$$

The Lévy flight distribution, denoted as Levy(Dim), is calculated as follows:

(8) $$\mathrm{Levy}(\mathrm{Dim})=\;s\;\times \frac{u\;\times \;\sigma }{{\left|v\right|}^{\frac{1}{\;\eta }}}$$

where *u* and *v* are random variables uniformly distributed in [0,1], and *s* = 0.01, *η* = 1.5. The parameter *σ* is defined as:

(9) $$\sigma \;=\;{\left(\frac{\Gamma (1+\eta )\;\times \;\sin\left(\frac{\pi \eta }{2}\right)}{\Gamma \left(\frac{1+\eta }{2}\right)\;\times \;\eta \;\times \;2\left(\frac{\eta -1}{2}\right)}\right)}^{\frac{1}{\eta }}$$

In Equation (9), Γ  refers to the gamma function, and the parameter η is set to 1.5.

(ii) Escape behavior in the exploitation stage

In the escape-from-predator stage, the position of each individual is updated using either a camouflage-based move (C_1_) or a rapid-escape move (C_2_), as defined by Equation (10).

In the first strategy, a dynamic perturbation factor ${\left(1-\frac{t}{T}\right)}^{2}$ is introduced to adaptively balance exploration and exploitation during the search process. The position update combines guidance from the current best solution with stochastic perturbations generated by random vectors and candidate solutions. This adaptive mechanism enables flexible probabilistic switching between global exploration and local exploitation across different iterations. The updated position is retained only if it yields an improved fitness value.

(10) $$X_{i,j}^{\mathrm{new},E2}=\{\begin{array}{c} {C_{1}\;:\;X}_{\mathrm{best}}+\left({\left(1-\frac{t}{T}\right)}^{2}\right)\times X_{i,j}\times (2RB-1),\;if\;r\;\mathrm{and}<r_{i}\;\\ C_{2\;\;}:{\;X}_{i,j}+R_{2}\times {(X}_{\mathrm{random}}-Κ\times X_{i,j}),\;\mathrm{else} \end{array}$$

(11) $$X_{i}=\{\begin{array}{c} X_{i}^{\mathrm{new},E2},\;if\;F_{i}^{\mathrm{new},E2}<F_{i}\\ X_{i},\;\mathrm{else} \end{array}$$

Here, r is fixed at 0.5 to probabilistically control the switching between the two update strategies. The random vector $R_{2}$ is generated from a normal distribution with dimension 1×Dim, while $x_{\mathrm{random}}$ denotes a randomly selected candidate solution from the current iteration. The parameter K is obtained via uniform random sampling and represents a randomly selected integer value of either 1 or 2, enabling dynamic selection between alternative update mechanisms.

### 3.2. Enhanced Secretary Bird Optimization Algorithm

The Enhanced Secretary Bird Optimization Algorithm (ESBOA) is proposed to address the limitations of the standard SBOA for energy-efficient CH selection. The main objective of ESBOA is to enhance convergence stability and solution quality by strengthening two critical stages of optimization: population initialization and solution refinement. It is explained that the core updating operators of SBOA described in Section 3.1 are retained, while additional mechanisms are incorporated to enhance the overall search behavior.

During the initial iterations, insufficient population diversity may lead to premature convergence toward suboptimal CH configurations, resulting in unbalanced energy consumption and degraded network performance. Additionally, weak exploitation capability in later iterations may hinder effective refinement of promising CH candidates. By combining a chaos-based population initialization strategy and an Iterative Local Search (ILS) refinement mechanism, ESBOA addresses these limitations. By enhancing both global exploration and local exploitation, ESBOA is designed to generate well-distributed and energy-rich CH configurations that directly support balanced energy consumption and extended network operation.

#### 3.2.1. Adaptive Population Initialization

Population initialization plays a critical role in the performance of metaheuristic optimization algorithms, as it directly influences population diversity, convergence speed, and solution quality. An inadequately initialized population may lead to premature convergence toward local optima, whereas poorly distributed initial solutions may increase computational cost without proportional performance gains. Therefore, an effective initialization strategy is essential to balance exploration capability and computational efficiency.

To address the limitations of conventional random initialization, a chaos-based population initialization strategy is adopted to enhance population diversity during the early optimization stages. Chaotic maps [27] exhibit ergodicity, randomness, and sensitivity to initial conditions, which enable a more uniform and well-distributed coverage of the search space. These properties are particularly advantageous in high-dimensional optimization problems, where insufficient diversity may cause the search process to stagnate around suboptimal regions.

In this study, a logistic chaotic map [28] is employed to generate the initial population of the proposed ESBOA. The logistic map is defined as:

(12) $$x_{n+1}=\;\mu x_{n}(1-x_{n})$$

where $x_{n}$ denotes the n-th chaotic value constrained within the interval (0, 1) and μ is a control parameter that regulates the system dynamics, with μ∈(0,4]. The generated chaotic sequence is then mapped to the feasible search space to initialize the positions of secretary birds, providing a more diverse and uniformly distributed initial population compared to conventional random initialization.

#### 3.2.2. Local Search Mechanism

Metaheuristic algorithms are effective for global exploration but often exhibit limited local exploitation capability, particularly in the later stages of optimization, which may lead to premature convergence. To overcome this limitation, a local search mechanism is incorporated into the proposed ESBOA as a refinement step applied after the standard SBOA updating process, aiming to further improve the quality of promising solutions without altering the core SBOA operators.

In this study, an Iterative Local Search (ILS) [29] mechanism is employed to enhance solution quality by exploring the neighborhood of the current solution selected for refinement through controlled perturbations. Starting from a candidate solution obtained by the SBOA update, a neighboring solution is generated and evaluated. The newly generated solution replaces the current solution only if an improvement in fitness is achieved, thereby ensuring a greedy refinement strategy. The local perturbation is defined as:

(13) $$x_{nei}=\;x_{ils}\;+\;p\;(\mathrm{rand}\;(1,J)\;-\;r)s$$

where $x_{ils}$ represents the current solution selected as the starting point of the ILS refinement process. The parameter p denotes the disturbance factor that controls the strength of the applied perturbation. The operator rand (1,J) generates a random matrix with the same shape as the current solution, while r represents the disturbance value used to shift the perturbation range. The parameter s defines the neighborhood size and controls the magnitude of the local perturbation. Together, p and s control the step size and search scope of the ILS process, ensuring a balance between neighborhood exploration and local solution refinement. These parameters collectively determine the newly generated neighboring solution $x_{\mathrm{nei}}$.

The enhanced optimization mechanisms described in Section 3.2 focus on improving the search behavior of the standard SBOA by strengthening population diversity and local refinement capabilities. However, the effectiveness of ESBOA in addressing the energy-efficient CH selection problem fundamentally depends on how candidate solutions are evaluated during the optimization process. In clustered WSNs, CH selection is a multi-objective decision-making problem that must simultaneously consider residual energy, communication distance, and node degree to ensure balanced energy dissipation and prolonged network lifetime. Therefore, a problem-specific fitness function is required to explicitly guide the ESBOA search toward energy-efficient and well-balanced CH configurations. In the following subsection, a multi-criteria fitness function tailored to the energy-efficient CH selection problem is formulated, providing a direct link between the ESBOA optimization process and the clustering objectives in WSNs.

### 3.3. Multi-Criteria Fitness Function

To evaluate candidate solutions generated by the proposed ESBOA-based clustering protocol, a multi-criteria fitness function is formulated to explicitly assess the suitability of sensor nodes for CH selection. The fitness function is designed to guide the optimization process toward energy-efficient and well-balanced clustering decisions by minimizing overall energy consumption while maintaining efficient communication and load distribution within the network.

In this study, the fitness function jointly considers three key criteria: residual energy, distance to the base station, and node degree. Residual energy reflects the remaining energy level of candidate CH and is prioritized to prolong network lifetime. The distance to the base station represents the transmission cost associated with long-range communication, while node degree indicates the local node density around a candidate CH and contributes to balanced intra-cluster load distribution.

(14) $$f(x)=w_{1}\cdot \left(\frac{1}{E_{res}}\right)+w_{2}\cdot d_{BS}+w_{3}\cdot \left(\frac{1}{\mathrm{Deg}}\right)$$

where $E_{\mathrm{res}}$ denotes the residual energy of the selected CH candidates, $d_{\mathrm{BS}}$ represents the Euclidean distance between the CH and the base station, and Deg indicates the node degree. The weights coefficients $w_{1}$, $w_{2}$, and $w_{3}$ control the relative importance of each criterion in the fitness evaluation. Accordingly, the fitness value is minimized during the optimization process to obtain energy-efficient and well-balanced CH configurations.

### 3.4. Pseudocode of the Proposed ESBOA Method

The proposed ESBOA-based CH selection procedure starts by initializing the optimization parameters and generating an initial population using a logistic chaotic map to enhance solution diversity. At each iteration, candidate solutions representing CH configurations are evaluated using the multi-criteria fitness function described in Section 3.3. The population is then updated through the standard SBOA exploration and exploitation phases according to the iteration stage to maintain a balance between global and local search. To ensure feasible CH selection, each updated solution is repaired to satisfy clustering constraints, and the global best solution is further refined using the ILS mechanism to avoid premature convergence. The process continues until the termination condition is met, and the final global best solution is selected as the energy-efficient CH configuration.

The computational complexity of the proposed ESBOA-based CH selection is primarily influenced by the population size N, the maximum number of iterations T, and the problem dimensionality Dim, which corresponds to the number of candidate sensor nodes considered for CH selection. In each iteration, the algorithm performs population update operations based on the exploration and exploitation mechanisms of SBOA, followed by fitness evaluation using the multi-criteria objective function. These operations incur a computational cost of O(N⋅Dim) per iteration, resulting in an overall time complexity of O(T⋅N⋅Dim). The logistic chaotic map–based population initialization is executed only once at the beginning of the optimization process and introduces an additional cost of O(N⋅Dim), which does not affect the asymptotic complexity. Moreover, the embedded ILS mechanism is applied only to the global best solution in each iteration, incurring an additional cost of O(Dim) per iteration. Consequently, the overall computational complexity of ESBOA remains O(T⋅N⋅Dim), which is of the same order as that of the standard SBOA. Since the entire CH selection procedure is executed offline at the base station prior to network operation, the proposed method does not impose computational or energy burdens on sensor nodes during data transmission.

In addition to the asymptotic computational complexity, the overhead costs introduced by the proposed ESBOA are mainly associated with the chaos-based population initialization and the embedded ILS mechanism. The logistic chaotic map is executed only once during the initialization stage and replaces conventional random initialization; therefore, it does not introduce iterative overhead during the optimization process. The ILS mechanism is applied exclusively to the global best solution in each iteration rather than to the entire population, resulting in a limited and controlled overhead proportional to the problem dimensionality.

Compared with the standard SBOA, the additional overhead of ESBOA is marginal and does not alter the dominant computational cost. Moreover, population-based metaheuristics such as CPO and DBO also incur comparable per-iteration overhead due to population updates, fitness evaluations, and constraint handling. Consequently, ESBOA achieves improved solution quality and convergence stability with only minor additional overhead, making it computationally competitive with related swarm-based methods for energy-efficient CH selection in clustered WSNs.

**Figure 2:**
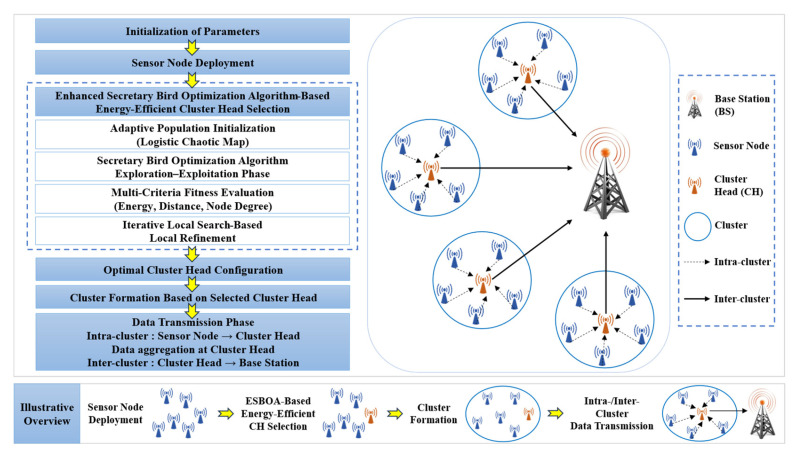
ESBOA-based energy-efficient CH selection and clustering workflow in WSNs.

Algorithm 1 explicitly integrates CH encoding, constraint enforcement, cluster member assignment, and multi-criteria energy-based fitness evaluation within the ESBOA framework, thereby ensuring that the optimization process is directly aligned with the objective of energy-efficient CH selection in WSNs. To provide a clearer overview of the proposed method and its integration within the clustering process, the overall framework of the ESBOA-based energy-efficient clustering protocol is presented in Figure 2.
**Algorithm 1** ESBOA-Based Energy-Efficient Cluster Head SelectionInput: Problem Setting (*Dim*, *ub*, *lb*, Pop_size (*N*), Max_Iter (*T*)) Output: Energy-efficient cluster head (CH) configuration 1: Encode each candidate solution as a CH configuration 2: Initialize the population using logistic chaotic map in Equation (12) 3: Evaluate fitness of each CH configuration using Equation (14) 4: Determine initial global best solution *x_best_*
5: **for** *t* = 1:*T* 6:  **for** *i* = 1:*N* 7:   **SBOA Exploration Phase:** 8:   **if** *t* < $\frac{1}{3}$ *T*
9:    Generate new CH configuration using Equation (2) 10:     Update solution using Equation (3) 11:   **else if** $\frac{1}{3}$ *T* < *t* < $\frac{2}{3}$ *T* 12:    Generate new CH configuration using Equation (4) 13:    Update solution using Equation (5) 14:   **else** 15:   Generate new CH configuration using Equation (6) 16:   Update solution using Equation (7) 17:   **end if** 18:   **SBOA Exploitation Phase:** 19:   **if r < 0.5** 20:   Generate new CH configuration using C_1_ in Equation (10) 21:   **else** 22:   Generate new CH configuration using C_2_ in Equation (10) 23:   **end if** 24:   Update solution using Equation (11) 25:   Repair solution to satisfy CH selection constraints 26:   Assign cluster members to the nearest CH 27:   Evaluate fitness of updated CH configuration 28:  **end for** 29:  Update global best CH configuration *x_best_* 30:  Apply Iterative Local Search to refine *x_best_* using Equation (13) 31:  Update *x_best_* if improved 32: **end for** 33: Return energy-efficient CH configuration *x_best_*.

The procedure begins with parameter initialization and sensor node deployment, followed by the initialization of an adaptive population that utilizes a logistic chaotic map to enhance solution diversity. The Secretary Bird Optimization Algorithm performs global exploration and exploitation to generate candidate CH configurations, which are evaluated using a multi-criteria energy-based fitness function that includes residual energy, distance to the base station, and node degree. Each candidate solution encodes a potential CH configuration, and constraint handling ensures that only feasible CH sets satisfying network requirements are retained during the optimization process. The iterative local search method is then used to improve the optimal CH configuration. The optimized set of CHs is used for cluster formation and intra/inter-cluster data transmission, thereby ensuring balanced energy dissipation and extended network lifetime in WSNs.

## 4. Results and Discussion

To evaluate whether the proposed ESBOA effectively addresses the energy-efficient CH selection problem, controlled simulations were conducted using a Python 3.11.9-based framework. The analysis aims to verify whether the CH configurations generated by ESBOA promote balanced energy dissipation, delay premature node failures, and sustain stable network performance over time. For a fair and unbiased comparison, ESBOA was evaluated against three representative metaheuristic baselines—standard SBOA [26], Crested Porcupine Optimizer (CPO) [21], and Dung Beetle Optimizer (DBO) [22]—under identical network configurations and the same first-order radio energy model. The validation is based on five key performance indicators directly associated with energy-aware CH selection: the number of alive nodes per round, residual energy, network lifetime, cumulative packets delivered to the base station, and lifetime performance under different deployment environments. Alive nodes and network lifetime quantify energy balancing capability, residual energy reflects load distribution efficiency, cumulative packets assess communication reliability, and deployment-based lifetime analysis verifies robustness and scalability. Collectively, these indicators provide comprehensive evidence of whether ESBOA improves CH selection quality compared with existing approaches.

### 4.1. Simulation Settings

This study uses the First-Order Radio Energy Model [30] to estimate the energy consumption associated with wireless communication in clustered WSN. This model is widely used in WSN research because it is simple and effective in identifying the main sources of energy dissipation during transmission, reception, and aggregation. It also ensures a realistic and consistent evaluation of energy-efficient CH selection. In this framework, the sensor node’s energy use depends on the electronic circuits used for sending and receiving data and the power amplification needed for wireless communication. Additionally, due to the data aggregation operations performed on the data collected from member nodes, the CH incurs additional energy costs. The adopted energy model incorporates these elements and provides a realistic representation of the energy costs associated with communication in clustered WSNs. Moreover, this model allows for a reasonable assessment of how various CH selection strategies affect overall energy efficiency.

The energy required to transmit an *m*-bit message over a distance *d* is given by:

(15) $$E_{Tx}\;\left(m,\;d\right)=\;\left\{\begin{array}{c} m\times E_{\mathrm{elec}}+m\times \varepsilon_{fs}\times d^{2},\;\;\mathrm{if}\;\;d<d_{0}\;\\ m\times E_{\mathrm{elec}}+m\times \varepsilon_{mp}\times d^{4},\;\;\mathrm{if}\;\;d\geq d_{0} \end{array}\right\}$$

where $E_{\mathrm{elec}}\;$ represents the energy consumed per bit for the transmitter or receiver circuitry, while $\varepsilon_{fs}$ and $\varepsilon_{mp}$ denote the amplification coefficients for the free space and multipath fading models, respectively.

The threshold distance *d*_0_ that determines the propagation model is computed as:

(16) $$d_{0}=\;\sqrt{\frac{\varepsilon_{fs}}{\varepsilon_{mp}}}\;$$

Meanwhile, the energy required to receive an *m*-bit message is computed as:

(17) $$E_{Rx}(m,\;d)=m\times E_{elec}$$

In clustered communication, CH incur additional energy consumption due to data aggregation. The aggregation energy is modeled as a constant energy cost per bit, denoted by $E_{\mathrm{DA}}$. For an m-bit message, the aggregation energy consumed at a CH is expressed as:

(18) $$E_{DA}(m)=m\times E_{DA}$$

All simulation parameters were kept identical across the compared algorithms to ensure an unbiased evaluation, as summarized in Table 1. This configuration allows the observed performance differences to be attributed directly to the quality of the CH selection process rather than to variations in network or energy model settings.

### 4.2. Homogeneous Network Model

**Figure 3:**
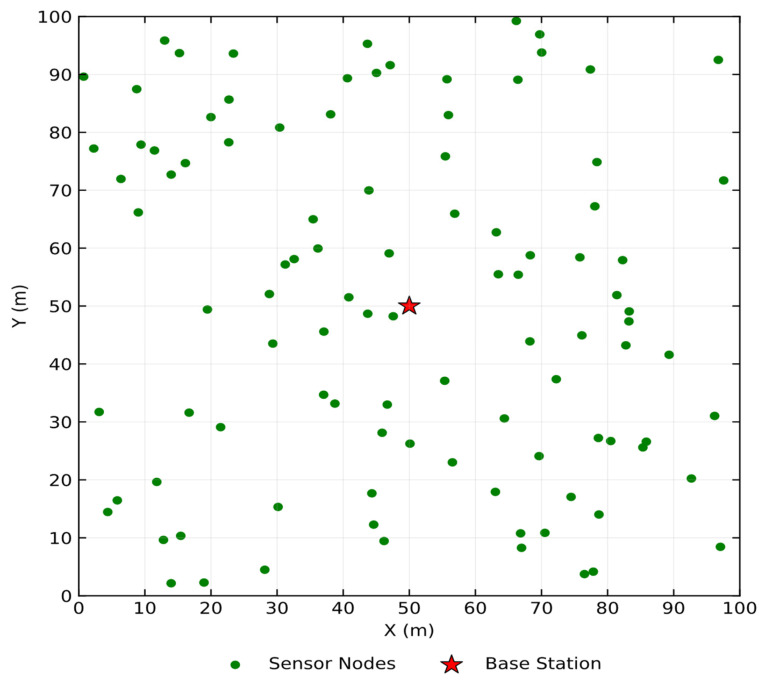
Homogeneous clustered WSN model.

A homogeneous WSN consisting of N sensor nodes is considered in this study. The sensor nodes are randomly deployed within a two-dimensional square monitoring area of size T×T, and their positions remain fixed after deployment. Each sensor node is assigned a unique identifier and is equipped with sensing, processing, and wireless communication capabilities. A single base station is assumed to be located at a fixed position within the network. Communication between sensor nodes and the base station is carried out using a cluster-based architecture. In each communication round, a subset of sensor nodes is selected as CH, while the remaining nodes act as cluster members. Each CH is responsible for collecting sensed data from its associated members, performing data aggregation, and forwarding the aggregated information to the base station. Figure 3 illustrates the homogeneous WSN deployment scenario and the corresponding clustered communication structure.

The network operates in discrete rounds, each consisting of a setup phase for CH selection and cluster formation, followed by a steady-state phase for data transmission. All sensor nodes are assumed to have identical initial energy levels and hardware characteristics, ensuring a homogeneous network environment. The energy of the base station is considered unlimited and therefore does not constrain network operation. This homogeneous network model provides a controlled and consistent environment for evaluating the effectiveness of different CH selection strategies, allowing performance differences to be attributed directly to the quality of the CH selection process.

#### 4.2.1. Alive Nodes Number

**Figure 4:**
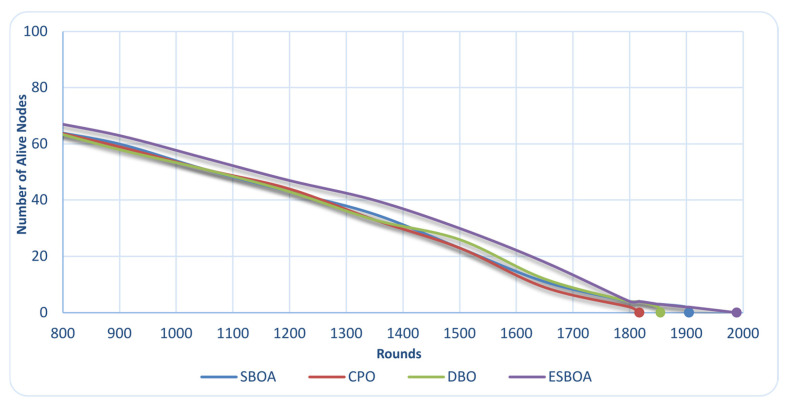
Comparison of the Number of Alive Nodes per Rounds between ESBOA, SBOA [26], CPO [21], and DBO [22].

Figure 4 illustrates the evolution of the number of alive nodes over successive communication rounds for a homogeneous WSN consisting of 100 sensor nodes. In this context, an alive node refers to a sensor node that retains sufficient residual energy to continue participating in sensing, intra-cluster communication, and data transmission to the base station. Therefore, the number of alive nodes serves as a direct indicator of how effectively the CH selection strategy balances energy consumption across the network. As shown in Figure 4, ESBOA consistently maintains more alive nodes compared to the baseline algorithms SBOA, CPO, and DBO throughout the simulation period. This improvement indicates that the CH configurations generated by ESBOA avoid selecting low-energy or poorly positioned nodes as CHs, thereby reducing premature energy depletion. By prioritizing nodes with higher residual energy and more favorable spatial characteristics, ESBOA achieves a more balanced energy dissipation pattern among sensor nodes.

The postponed decline in active nodes under ESBOA illustrates its capacity to avert premature node failures, typically resulting from imbalanced CH workloads and high energy expenditure at certain nodes. The preservation of an extensive array of active nodes fosters stable clustering configurations, enduring communication pathways, and uninterrupted data transmission to the base station. Thus, the noted enhancement in the preservation of living nodes offers definitive proof that the proposed ESBOA effectively tackles the energy-efficient CH selection issue by augmenting early-stage energy equilibrium and strengthening overall network stability.

#### 4.2.2. Residual Energy

**Figure 5:**
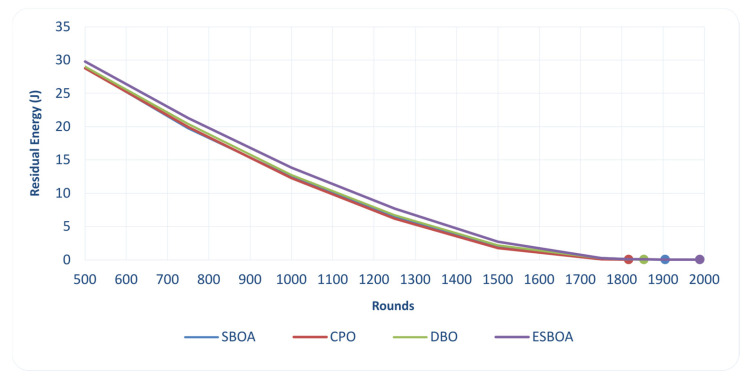
Comparison of the Residual Energy per Rounds between ESBOA, SBOA [26], CPO [21], and DBO [22].

Figure 5 shows the change curve of the residual energy in the whole network with different communication rounds. Residual energy is one of the important factors used to measure the performance of energy consumption on clustered WSNs, which directly represents how well a CH selection strategy distributes communications and aggregation workloads among sensor nodes. In Figure 5, it can be seen that ESBOA has higher residual energy than all the benchmark algorithms, SBOA, CPO, and DBO, in the whole simulation period. This phenomenon suggests that the energy-oriented CH selection mechanism of ESBOA can effectively prevent some nodes, such as those selected multiple times, from consuming their own excessive energy. Through favoring higher residual energy nodes with suitable spatial properties, ESBOA assists in shaping a balanced distribution of energy consumption through the network.

Furthermore, the less amplitude and slower decrease in residual energy under ESBOA is due to a stable energy dissipation mechanism, indispensable for long-term network working. Such balanced energy consumption not only lessens the occurrence of energy hotspots and early node failures but also prolongs network connectivity. Therefore, our residual energy analysis gives direct proof that ESBOA contributes to increased energy efficiency by making better CH selection decisions, resulting in prolonged network lifetime.

#### 4.2.3. Network Lifetime

**Figure 6:**
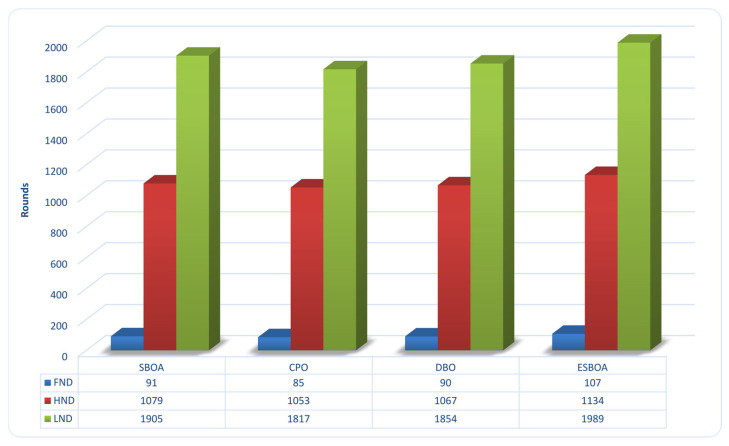
Comparison of Network Lifetime Metrics (FND, HND, and LND) between ESBOA, SBOA [26], CPO [21], and DBO [22].

The network lifetime metrics for each evaluated algorithm are compared in Figure 6 with respect to First Node Death (FND), Half Node Death (HND), and Last Node Death (LND). The proposed ESBOA clearly improves network sustainability by continuously outperforming the baseline approaches (SBOA, CPO, and DBO) across all three indicators. In particular, ESBOA shows a more balanced early energy consumption by delaying the first node failure to 107 rounds as opposed to 91, 85, and 90 rounds for SBOA, CPO, and DBO, respectively. This advantage is further demonstrated by the HND and LND metrics, where ESBOA outperforms the nearest rival, SBOA, which achieves 1079 (HND) and 1905 (LND) rounds, by extending network operation to 1134 and 1989 rounds. Because of its improved population initialization and local search mechanisms, which allow the selection of well-distributed and energy-rich CH, ESBOA has a longer lifetime. As a result, energy dissipation is distributed more evenly among sensor nodes, which lowers the risk of early node failures and prolongs stable network connectivity.

Beyond the absolute lifetime extension, the improvements observed in FND, HND, and LND collectively indicate that ESBOA achieves a more gradual and stable energy depletion pattern compared with the baseline algorithms. The delayed FND suggests that ESBOA effectively avoids selecting low-energy nodes as CH during the early rounds, while the extended HND reflects a balanced energy consumption among a larger portion of the network. Moreover, the significant gain in LND demonstrates that the proposed method sustains network operability for a longer duration by preventing premature exhaustion of critical nodes. This behavior confirms that the combined effects of chaotic population initialization and ILS enable ESBOA to maintain both early-stage robustness and long-term stability, which are essential for reliable clustered WSNs operation.

#### 4.2.4. Cumulative Packets to Base Station

**Figure 7:**
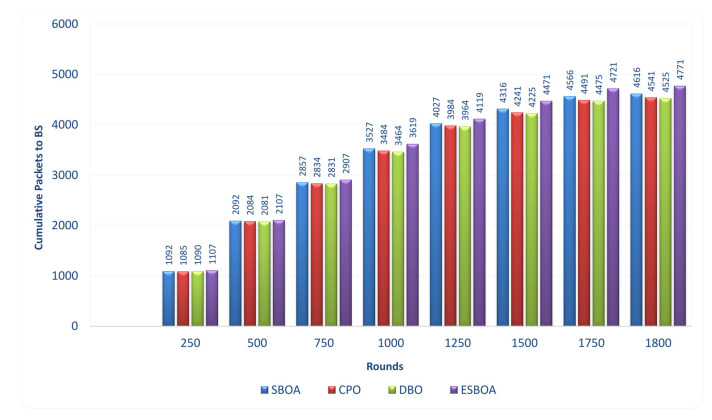
Comparison of Cumulative Packets to BS per Rounds between ESBOA, SBOA [26], CPO [21], and DBO [22].

The evolution of cumulative data packets received at the base station across simulation rounds is evaluated to assess the data delivery performance of the compared algorithms. According to the findings, ESBOA continuously achieves the highest cumulative packet count at every observation point, demonstrating superior throughput and long-term network operation. Due to its capacity to sustain more active nodes and stable cluster structures, ESBOA delivers more packets than SBOA, CPO, and DBO in the early and mid-simulation stages. The performance gap widens as the number of rounds increases, with ESBOA reaching the highest cumulative packets at the simulation’s conclusion while the baseline methods grow more slowly because of earlier node failures and decreased data transmission capability. This result indicates that the selected CH remain energy-efficient for longer periods, allowing continuous data forwarding to the base station. The energy-efficient CH selection of ESBOA, which balances energy consumption among nodes, reduces packet loss, and avoids premature network disconnection, is responsible for this improvement. As a result, ESBOA improves data delivery efficiency and also guarantees more reliable and continuous communication with the base station throughout the network’s lifetime. Figure 7 illustrates the cumulative number of data packets received at the base station as a function of simulation rounds for ESBOA, SBOA, CPO, and DBO.

In addition to achieving higher cumulative packet delivery, the sustained growth of cumulative packets in ESBOA indicates a more resilient data delivery process throughout the network lifetime. The consistently higher slope of the ESBOA curve reflects stable cluster operation and reduced packet loss, particularly in later simulation rounds when baseline methods experience accelerated node depletion. This behavior suggests that ESBOA increases throughput and maintains reliable end-to-end communication by preserving active CH and preventing frequent re-clustering events. Consequently, the observed improvement in cumulative packets confirms that the energy-efficient CH selection of ESBOA directly translates into enhanced network throughput and prolonged data reporting capability under long-term operation.

#### 4.2.5. Network Lifetime in Different Environments

To evaluate the robustness and adaptability of the proposed ESBOA under varying network scales, simulation experiments were conducted across multiple environmental scenarios with increasing monitoring area sizes, namely 100 × 100 m^2^, 200 × 200 m^2^, and 300 × 300 m^2^. These scenarios represent different levels of network complexity and communication distance, which directly affect energy consumption and node survivability. Higher FND, HND, and LND values show that ESBOA consistently outperforms the baseline algorithms in all scenarios in terms of network lifetime. By efficiently choosing well-distributed and energy-rich CH, ESBOA maintains a clear performance advantage even though an increase in the monitoring area generally results in a reduction in network lifetime due to longer transmission distances and higher energy expenditure. This behavior indicates that the proposed CH selection strategy remains effective under different deployment scales, allowing ESBOA to sustain stable network operation and balanced energy consumption in more challenging environments.

According to the LND values reported in Table 2, ESBOA achieves lifetime improvements of 4.4%, 2.9%, and 13.1% in Scenarios 1–3, respectively, compared with the standard SBOA. These scenario-wise results collectively explain the reported 3–13% improvement range and further confirm the consistent advantage of ESBOA over other metaheuristic baselines across different deployment environments.

## 5. Conclusions and Future Work

The proposed ESBOA improves energy-efficient CH selection in clustered WSNs by reinforcing two critical optimizer components, population initialization and solution refinement, while keeping the CH decision explicitly guided by a multi-criteria fitness function that considers residual energy, distance to the base station, and node degree. Using logistic chaotic map–based initialization increases early-stage solution diversity, and the embedded ILS strengthens exploitation to mitigate premature convergence, yielding more stable CH configurations and more balanced energy dissipation across nodes. Comparative simulations under identical settings against SBOA, CPO, and DBO demonstrate that ESBOA consistently preserves more alive nodes, maintains higher residual energy, increases cumulative packets delivered to the base station, and extends lifetime indicators FND, HND, and LND. In particular, ESBOA extends the network lifetime in terms of last node death (LND) by 4.4%, 2.9%, and 13.1% across the evaluated deployment scenarios compared to the standard SBOA, collectively corresponding to an overall improvement range of approximately 3–13%. These results confirm that the proposed enhancements effectively address the energy-efficient CH selection problem and translate into measurable network-level performance improvements rather than optimizer-only gains.

Despite these advantages, the current study is limited to homogeneous network scenarios and single-hop CH to base station communication under simulation-based evaluation. Future work will focus on integrating multi-hop CH to base station routing and adaptive constraint-handling mechanisms to further reduce long-range transmission costs, perform sensitivity and robustness analyses of ESBOA hyperparameters and fitness weights, and extend the evaluation toward real-world deployment and hardware-platform–based testbed validation under practical operating conditions.

## Figures and Tables

**Table 1:** Simulation parameters.

Parameters	Value
Network Field	(100, 100)
Number of nodes	100
Initial Energy (Eo)	0.5 J/node
Transmitter electronics (E_TX-elec_)	50 nJ/bit
Receiver electronics (E_RX-elec_)	50 nJ/bit
Packet Size (m)	4000 bits
$\mathrm{Energy}\;\mathrm{dissipation}\text{:}\;\mathrm{free}\;\mathrm{space}\;\mathrm{model}\;(\varepsilon_{fs}$) if d < d_0_	10 pJ/bit/m^2^
$\mathrm{Energy}\;\mathrm{dissipation}\text{:}\;\mathrm{power}\;\mathrm{amplifier}\;(\varepsilon_{mp}$) if d > d_0_	0.0013 pJ/bit/m^4^
Data Aggregation (E_da_)	5 nJ/bit
Probability of CH (p)	0.05

**Table 2:** Comparison of Network Lifetime in Different Environments.

Scenario	Protocol	FND	HND	LND
Scenario 1	CPO [21]	85	1053	1817
	DBO [22]	90	1067	1854
	SBOA [26]	91	1079	1905
	ESBOA	107	1134	1989
Scenario 2	CPO [21]	69	479	1021
	DBO [22]	71	587	1082
	SBOA [26]	72	676	1484
	ESBOA	105	678	1527
Scenario 3	CPO [21]	66	300	698
	DBO [22]	67	319	701
	SBOA [26]	70	361	1030
	ESBOA	100	371	1165

## Data Availability

The data presented in this study are available on request from the corresponding author.

## Abbreviations

The following abbreviations are used in this manuscript:

WSNs	Wireless Sensor Network
BS	Base Station
CH	Cluster Head
SBOA	Secretary Bird Optimization Algorithm
ESBOA	Enhanced Secretary Bird Optimization Algorithm
FND	First Node Death
HND	Half Node Death
LND	Last Node Death
